# Glucocorticoid Receptor Antagonist Mifepristone Does Not Alter Innate Anxiety-Like Behavior in Genetically-Selected Marchigian Sardinian (msP) Rats

**DOI:** 10.3390/ijms22063095

**Published:** 2021-03-18

**Authors:** Valentina Vozella, Bryan Cruz, Luis A. Natividad, Federica Benvenuti, Nazzareno Cannella, Scott Edwards, Eric P. Zorrilla, Roberto Ciccocioppo, Marisa Roberto

**Affiliations:** 1 Department of Molecular Medicine, The Scripps Research Institute, La Jolla, CA 92037, USA; vvozella@scripps.edu (V.V.); bcruz@scripps.edu (B.C.); ezorrill@scripps.edu (E.P.Z.); 2 College of Pharmacy, The University of Austin, Austin, TX 78712, USA; Luis.Natividad@austin.utexas.edu; 3 School of Pharmacy, Pharmacology Unit, University of Camerino, 62032 Camerino, Italy; federica.benvenuti@unicam.it (F.B.); nazzareno.cannella@unicam.it (N.C.); roberto.ciccocioppo@unicam.it (R.C.); 4 Department of Physiology, Louisiana State University, Health Sciences Center, New Orleans, LA 70112, USA; sedwa5@lsuhsc.edu

**Keywords:** anxiety, stress, sleep disturbances, hyperarousal, mifepristone, glucocorticoid receptor antagonist, alcohol-preferring rats

## Abstract

Marchigian Sardinian alcohol-preferring (msP) rats serve as a unique model of heightened alcohol preference and anxiety disorders. Their innate enhanced stress and poor stress-coping strategies are driven by a genetic polymorphism of the corticotropin-releasing factor receptor 1 (*CRF1*) in brain areas involved in glucocorticoid signaling. The activation of glucocorticoid receptors (GRs) regulates the stress response, making GRs a candidate target to treat stress and anxiety. Here, we examined whether mifepristone, a GR antagonist known to reduce alcohol drinking in dependent rats, decreases innate symptoms of anxiety in msPs. Male and female msPs were compared to non-selected Wistar counterparts across three separate behavioral tests. We assessed anxiety-like behavior via the novelty-induced hypophagia (NIH) assay. Since sleep disturbances and hyperarousal are common features of stress-related disorders, we measured sleeping patterns using the comprehensive lab monitoring system (CLAMS) and stress sensitivity using acoustic startle measures. Rats received an acute administration of vehicle or mifepristone (60 mg/kg) 90 min prior to testing on NIH, acoustic startle response, and CLAMS. Our results revealed that both male and female msPs display greater anxiety-like behaviors as well as enhanced acoustic startle responses compared to Wistar counterparts. Male msPs also displayed reduced sleeping bout duration versus Wistars, and female msPs displayed greater acoustic startle responses versus male msPs. Importantly, the enhanced anxiety-like behavior and startle responses were not reduced by mifepristone. Together, these findings suggest that increased expression of stress-related behaviors in msPs are not solely mediated by acute activation of GRs.

## 1. Introduction

Dysregulation of the hypothalamic-pituitary-adrenal (HPA) axis is a core feature of alcohol use disorder (AUD) and stress-related comorbidities [1]. Exposure to stressors initiates the activation of the HPA axis, which results in an increased release of corticotropin-releasing factor (CRF) from specific subnuclei of the hypothalamus, such as the paraventricular nucleus (PVN) [2]. Activation of corticotropin-releasing factor receptor 1 (CRF1) through CRF stimulates the release of adrenocorticotropic hormone from the anterior pituitary and subsequent glucocorticoid secretion from the adrenal glands. This systemic stress response is then terminated through a negative feedback process on the HPA axis when glucocorticoids bind to glucocorticoid receptors (GRs) [3], which are enriched in the hypothalamic PVN [4]. Furthermore, GR activity also impacts central brain stress circuitry, including major regulation of the amygdala and prefrontal cortex [5], to modulate cognition and negative affective states [6,7,8,9]. Thus, GR is considered to play an essential role in modulating both adaptive and maladaptive stress-associated behaviors [10].

Genetically-selected Marchigian Sardinian alcohol-preferring (msP) rats have been extensively characterized as a model of both enhanced alcohol preference and negative affective phenotypes [11,12]. The msP rats carry a unique mutation driven by two single nucleotide polymorphisms at the *CRF1* locus, leading to CRF1 receptor overexpression in areas of the brain associated with negative affect such as the amygdala [13,14,15,16,17,18]. This mutation causes innate hyperactivity of the CRF/CRF1 system, which correlates with excessive alcohol drinking [15], heightened stress sensitivity, potentiated negative affect, and behavioral alterations that possibly resemble post-traumatic stress disorder (PTSD) traits [14]. Ethanol drinking in msP rats is thought to be motivated by negative reinforcement, modeling the drinking behavior of a subpopulation of individuals who drink for tension relief and self-medication purposes [16,19].

Recently, we found that male msP rats display increased GR phosphorylation at serine 232, a site that is functionally associated with higher transcriptional activity, in the central nucleus of the amygdala (CeA) [20]. This is consistent with several reports demonstrating that GR phosphorylation is also increased in the CeA of alcohol-dependent rats during acute withdrawal [21]. Emerging evidence has shown that mifepristone, a potent GR and progesterone receptor (PR) antagonist with a higher binding affinity than the endogenous ligands [22], reliably reduces alcohol self-administration in dependent rats [21,23,24] and suppresses yohimbine stress-induced reinstatement of alcohol-seeking [25]. Furthermore, mifepristone has been clinically validated in a human laboratory model of craving and found to reduce the number of drinks per week in abstinent alcohol-dependent volunteers [21].

Mifepristone has also been used as a pharmacological tool to test the role of GR in several models of stress-induced anxiety-like behaviors. For instance, intracerebroventricular infusion of mifepristone prior to a restraint procedure abolished stress-induced anxiety-like behavior [26]. Systemic administration of mifepristone also decreased negative affect produced by chronic stress in mice with high-trait anxiety [27]. The functional effects of mifepristone as an anxiety-alleviating agent are mixed since other reports demonstrate that this drug produces no restorative changes to stress in a mouse model that lacks stress coping mechanisms [28].

Emerging clinical research suggests that there are sex differences in alcohol consumption and dependence, and evidence shows that the prevalence of alcohol consumption as a coping strategy to attenuate negative affective states (e.g., anxiety, depression, stress, and isolation) is higher in women than men. Similarly, preclinical studies have reported that female msP rats consume higher amounts of alcohol when compared to males [12]. Noteworthy, Borruto et al. [12] demonstrated that voluntary 10% alcohol drinking reduced elevated plus maze (EPM) anxiety-like behavior in male, but not in female msP rats. These observations point to the possibility that alcohol drinking in male and female msPs is motivated by different forms of anxiety (i.e., generalized anxiety versus stress-induced inability to engage in stress coping). Based on these observations, it is crucial to assess the potential anxiolytic effects of GR antagonism in both sexes.

In the present study, we explored whether acute systemic administration of the non-selective glucocorticoid receptor antagonist mifepristone reduces the inherited high stress responses and anxiety-like behaviors in msPs versus non-selected control Wistar rats. Specifically, we assessed male and female rats in a battery of tests that captures different anxiety-related features. We employed novelty-induced hypophagia (NIH) to test anxiety-like behaviors under novel environmental conditions. Since sleep disturbances are a hallmark of anxiety and stress-related disorders (e.g., PTSD), we examined whether genotypic and sex differences may alter diurnal sleep maintenance. Finally, we utilized stronger stress-sensitive measures such as high-intensity acoustic signals to assess startle responses. Moreover, we tested the hypothesis that GR signaling would play a key role in the regulation of these behaviors by examining the efficacy of the GR antagonist mifepristone.

## 2. Results

### 2.1. Effects of Mifepristone on Anxiety-Like Behavior

**Figure 1:**
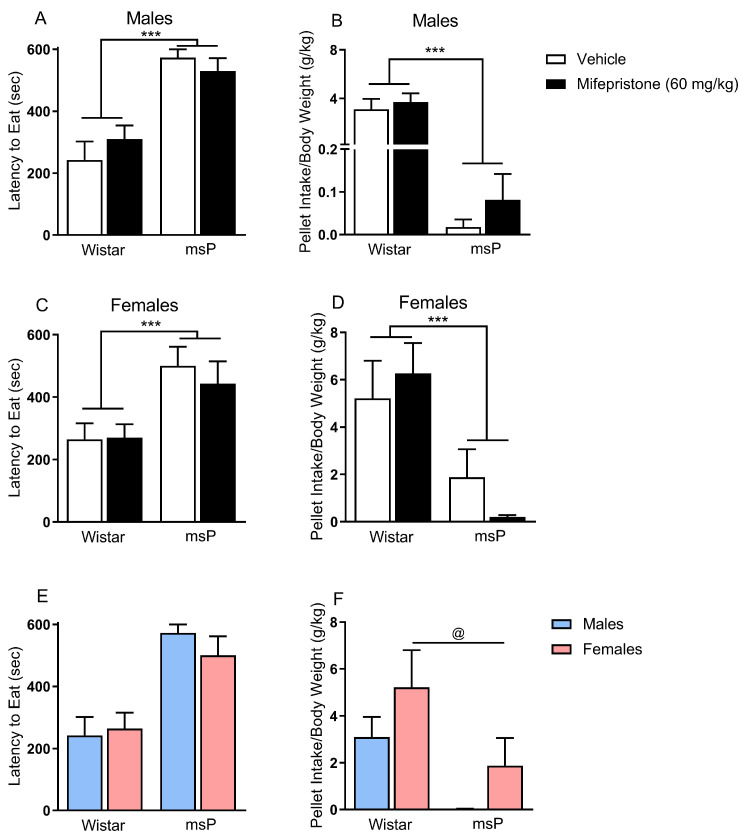
Effect of glucocorticoid receptor (GR) antagonist mifepristone on novelty-induced hypophagia (NIH) in both male and female Wistar and msP rats. Mifepristone (60 mg/kg) was injected intraperitoneally, and rats were subjected to the NIH test after 90 min. Male and female msP rats displayed higher anxiety-like behavior during NIH that was not reduced by mifepristone. (**A**) latency to eat chocolate pellets, (**B**) pellet intake in vehicle (*n* = 9) or mifepristone-treated (*n* = 10) male Wistar rats and vehicle (*n* = 13) or mifepristone-treated (*n* = 12) male msP rats. (**C**) latency to eat chocolate pellets, (**D**) pellet intake in vehicle (*n* = 9) or mifepristone-treated (*n* = 9) female Wistar rats and vehicle (*n* = 8) or mifepristone-treated (*n* = 10) female msP rats. (**E**) latency to eat chocolate pellets, (**F**) pellet intake in male Wistar (*n* = 9) and male msP (*n* = 13) rats, female Wistar (*n* = 9) and female msP (*n* = 8) rats. Results are expressed as mean ± SEM. Two-way ANOVA followed by Fisher’s LSD protected post hoc tests when an interaction between variables occurred. Main effect of genotype, *** *p* ≤ 0.001. Main effect of sex, ^@^ *p* ≤ 0.05.

To determine whether acute mifepristone administration reduces anxiety-like behavior in a genotype-specific manner, male and female Wistar and msP rats were pre-exposed to palatable chocolate pellets and then tested under novel environmental conditions using the novelty-induced hypophagia procedure. We found that male msPs in general displayed a greater latency to eat under novelty stress conditions as compared to male Wistar rats, main effect of genotype F_(1,40)_ = 42.52, *p =* 0.001 (Figure 1A). Male msPs also displayed lower overall pellet intake during novelty stress as compared to male Wistar rats, main effect of genotype F_(1,40)_ = 49.48, *p =* 0.0001 (Figure 1B). Interestingly, a single systemic mifepristone administration did not affect the latency to eat or the intake of chocolate pellets in male msPs, suggesting that the enhanced anxiety-like phenotype in msPs is not ameliorated by an acute administration of GR antagonist mifepristone. Similar to males, we found that female msPs displayed an increase in the latency to eat chocolate pellets under novelty stress conditions as compared to Wistar rats, main effect of genotype F_(1,32)_ = 12.10, *p =* 0.001 (Figure 1C). Female msPs also displayed lower overall pellet intake under novelty stress conditions relative to their Wistar counterparts, main effect of genotype F_(1,32)_ = 16.69, *p* = 0.0001 (Figure 1D). Importantly, mifepristone administration did not affect the latency to eat or intake of chocolate pellets in female msPs, suggesting that the enhanced anxiety-like phenotype observed in female msPs also is not ameliorated by acute exposure to the GR antagonist.

To further examine the contribution of sex differences in promoting anxiety-like behavior (Figure 1E,F), we also compared male versus female rats within each genotype as a function of sex, regardless of mifepristone treatment. We found that males displayed lower levels of pellet intake as compared to females regardless of genotype, suggesting that males displayed greater vulnerability produced by novelty stress, main effect of sex F_(1,35)_ = 4.91, *p* = 0.048 (Figure 1F).

### 2.2. Effects of Mifepristone on Sleep Disturbances

**Figure 2:**
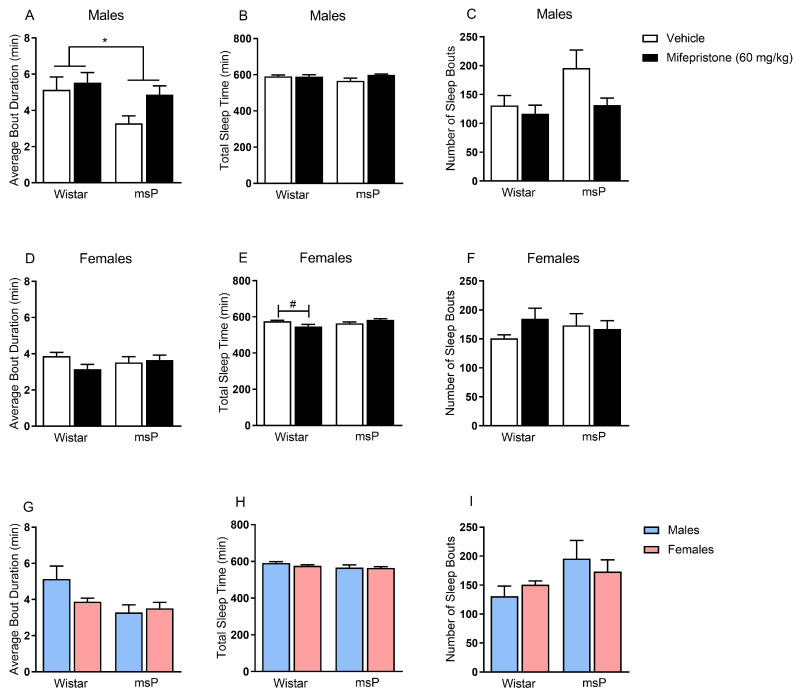
Effect of glucocorticoid receptor (GR) antagonist mifepristone on sleep disturbances in both male and female Wistar and msP rats. Mifepristone (60 mg/kg) was injected intraperitoneally, and rats were subjected to the comprehensive lab animal monitoring system (CLAMS) test after 90 min. Mifepristone had no effect on ameliorating sleep disturbances. (**A**) average bout duration, (**B**) total sleep time, (**C**) number of sleep bouts in vehicle (*n* = 8) or mifepristone-treated (*n* = 8) male Wistar rats and vehicle (*n* = 7) or mifepristone-treated (*n* = 8) male msP rats. (**D**) average bout duration, (**E**) total sleep time, (**F**) number of sleep bouts in vehicle (*n* = 8) or mifepristone-treated (*n* = 8) female Wistar rats and vehicle (*n* = 8) or mifepristone-treated (*n* = 8) female msP rats. (**G**) average bout duration, (**H**) total sleep time, (**I**) number of sleep bouts in male Wistar (*n* = 8) and male msP (*n* = 7) rats, female Wistar (*n* = 8) and female msP (*n* = 8) rats. Results are expressed as mean ± SEM. Two-way ANOVA followed by Fisher’s LSD protected post hoc tests when the interaction between variables occurred. Main effect of genotype, * *p* ≤ 0.05. Post hoc test revealed significant differences between treatments, ^#^ *p* ≤ 0.05.

To examine whether mifepristone administration restores sleep disturbances produced by heightened stress, we first assessed diurnal sleeping patterns in male and female Wistar and msP rats. We found that male msPs, in general, displayed a shorter average bout duration when compared to male Wistar rats, main effect of genotype F_(1,27)_ = 4.92, *p* = 0.035 (Figure 2A). Interestingly, mifepristone administration did not restore the reduced average bouts’ duration in male msPs suggesting that the interrupted sleep observed in male geneticallyselected msPs is not ameliorated by a single administration of GR antagonist. Importantly, no genotype differences or acute mifepristone effects were observed in total sleep time (Figure 2B) or number of sleep bouts (Figure 2C).

In females, we observed that mifepristone administration decreased total sleep time in a genotype-specific manner, drug × genotype interaction F_(1,28)_ = 9.36, *p* = 0.005 (Figure 2E). Specifically, Wistar rats that received mifepristone displayed a significant reduction in total sleep time as compared to vehicle-treated controls (*p* = 0.015). The latter effect was only observed in non-selected Wistar rats suggesting that mifepristone does not influence sleep patterns in genetically-selected msP rats. Importantly, there were no changes observed in average bout duration (Figure 2D) or number of sleep bouts (Figure 2F).

To further delineate the contribution of sex in promoting sleep disturbances, we compared male versus female rats within each genotype as a function of sex, regardless of mifepristone treatment. Here, we found no sex-dependent changes in average bout duration (Figure 2G), total sleep time (Figure 2H), or number of sleep bouts (Figure 2I).

### 2.3. Effects of Mifepristone on Hyperarousal States

**Figure 3:**
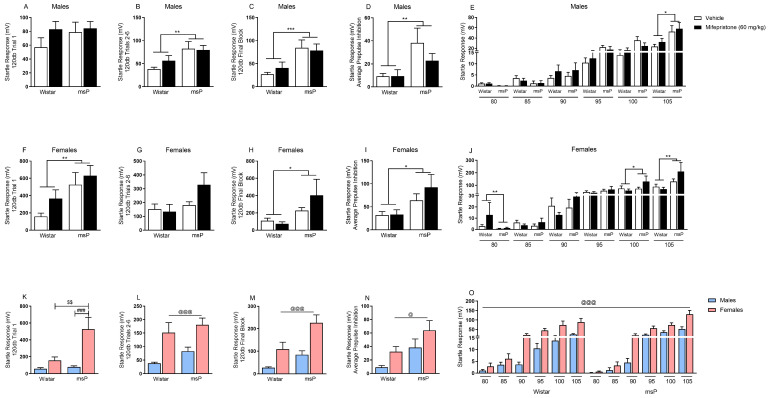
Effect of glucocorticoid receptor (GR) antagonist mifepristone on hyperarousal states in both male and female Wistar and msP rats. Mifepristone (60 mg/kg) was injected intraperitoneally, and rats were subjected to the acoustic startle response test after 90 min. Mifepristone had no effect on ameliorating the startle response to acoustic stimuli. (**A**) 120 dB trial 1 startle response, (**B**) 120 dB trials 2–6 startle response, (**C**) 120 dB final block startle response, (**D**) average prepulse inhibition startle response, (**E**) 80–105 dB startle responses in vehicle (*n* = 8) or mifepristone-treated (*n* = 8) male Wistar rats and vehicle (*n* = 6) or mifepristone-treated (*n* = 8) male msP rats. (**F**) 120 dB trial 1 startle response, (**G**) 120 dB trials 2–6 startle response, (**H**) 120 dB final block startle response, (**I**) average prepulse inhibition startle response, (**J**) 80–105 dB startle responses in vehicle (*n* = 8) or mifepristone-treated (*n* = 8) female Wistar rats and vehicle (*n* = 6) or mifepristone-treated (*n* = 8) female msP rats. (**K**) 120 dB trial 1 startle response, (**L**) 120 dB trials 2–6 startle response, (**M**) 120 dB final block startle response, (**N**) average prepulse inhibition startle response, (**O**) 80–105 dB startle responses in male Wistar (*n* = 8) and male msP (*n* = 6) rats, female Wistar (*n* = 8) and female msP (*n* = 8) rats. Results are expressed as mean ± SEM. Two-way ANOVA followed by Fisher’s LSD protected post hoc tests when interaction between variables occurred. For repeated stimulus intensities (**E,J,O**), a mixed model three-way ANOVA was used. Main effect of genotype, * *p* ≤ 0.05, ** *p* ≤ 0.01, *** *p* ≤ 0.001. Main effect of sex, ^@@@^ *p* ≤ 0.001, ^@^ *p* < 0.05. Post hoc test revealed significant differences between genotypes, ^$$^ *p* ≤ 0.01. Post hoc test revealed significant differences between sexes, ^###^ *p* ≤ 0.001.

Since mifepristone did not reduce the heightened anxiety-like behavior observed in genetically-selected msPs (Figure 1A–D), we employed stronger, more stress-sensitive measures often necessary for this drug to induce changes [25]. We used stress-sensitive procedures that capture startle reflexive responses following sound stimuli across various intensity trials. In males, we found that msPs in general displayed higher startle responses when compared to male Wistar rats during the 120 dB trials 2–6, main effect of genotype F_(1,26)_ = 10.56, *p* = 0.003 (Figure 3B) and 120 dB final block, main effect of genotype F_(1,26)_ = 14.51, *p* = 0.001 (Figure 3C). Furthermore, male msPs also displayed higher average prepulse inhibition when compared to male Wistar rats, main effect of genotype F_(1,26)_ = 9.32, *p* = 0.005 (Figure 3D). A three-way ANOVA across the sequence of various levels of intensities revealed an intensity × genotype interaction, F_(5,130)_ = 9.25, *p* = 0.001 (Figure 3E). Specifically, male msPs displayed a higher startle response at 105 dB stimulus when compared to male Wistar rats (*p* = 0.027) (Figure 3E). Interestingly, mifepristone administration did not reduce the genotypic differences in the enhanced startle response or prepulse inhibition behavior, suggesting that GR antagonism does not mitigate the msP sensitivity to these stress phenotypes.

In females, we observed that msPs in general displayed higher startle responses when compared to female Wistar rats during the 120 dB trial 1, main effect of genotype F_(1,28)_ = 8.89, *p* = 0.006 (Figure 3F) and 120 dB final block, main effect of genotype F_(1,28)_ = 5.37, *p* = 0.028 (Figure 3H). Female msPs also displayed higher average prepulse inhibition when compared to their counterpart Wistar rats, main effect of genotype F_(1,28)_ = 7.14, *p* = 0.012 (Figure 3I). Furthermore, a three-way ANOVA across the various sound intensities (i.e., 80–105 dB), revealed an intensity × genotype interaction F_(5,140)_ = 4.88, *p* = 0.026 (Figure 3J). Specifically, female msPs displayed higher startle responses at both the 100 dB (*p* = 0.011) and 105 dB stimuli (*p* = 0.006) when compared to female Wistar rats. Importantly, mifepristone administration did not reduce the enhanced startle response or prepulse inhibition suggesting that the heightened sensitivity phenotype observed within female genetically-selected msPs is not acutely ameliorated by the GR antagonist.

To examine the unique role of sex in promoting heightened stress sensitivity regardless of glucocorticoid blockade, we compared male versus female rats within each genotype as a function of sex. We found that female msPs displayed an increase in startle response during the 120 dB trial 1 when compared to female Wistar rats (Figure 3K). The two-way ANOVA revealed a sex × genotype interaction F_(1,26)_ = 4.81, *p* = 0.037, and the post hoc analysis showed that these effects were attributable to the different genotypes in a within females comparison (*p* = 0.002) and to the sex in a within msPs comparison (*p* = 0.001; Figure 3K). Interestingly, regardless of genotype, females overall displayed higher startle responses when compared to males during the 120 dB trials 2–6, main effect of sex F_(1,26)_ = 18.14, *p* = 0.0001 (Figure 3L) and during 120 dB final block, main effect of sex F_(1,26)_ = 18.20, *p* = 0.0001 (Figure 3M), as well as higher average prepulse inhibition, main effect of sex F_(1,26)_ = 5.61, *p* = 0.025 (Figure 3N). Lastly, females in general displayed higher startle responses across 80–105 dB series of intensities when compared to males, main effect of sex F_(1,26)_ = 20.02, *p* = 0.0001 and intensity × sex interaction F_(5,130)_ = 12.66, *p* = 0.0001 (Figure 3O). Taken together, these data suggest that females generally show higher startle susceptibility to sound stress than male rats.

## 3. Discussion

Increased anxiety, stress sensitivity, and an impaired ability to cope with stress are comorbid and, in some cases, promote the development of AUD, whereas in other cases, they are the consequences of excessive drinking and a reflection of alcohol dependence. Efficacious therapeutic interventions to alleviate these comorbid pathologies still lack [29], particularly for drugs that normalize glucocorticoid and other stress-related systems. Prior work has revealed that mifepristone, a non-selective GR antagonist, reliably reduces alcohol drinking and seeking in alcohol-dependent Wistar rats and humans [21,25]. As a rodent line that displays enhanced motivation for alcohol drinking and innate negative affect, msPs were tested on a battery of anxiety-related behavioral tests. We hypothesized that mifepristone would alleviate innate anxiety-related behaviors in msP rats versus non-selected Wistar counterparts. In summary, we found that male and female msPs display greater anxiety-like behaviors as compared to Wistars when tested in the NIH paradigm. In addition, while male msPs but not female msPs displayed lower average bout duration during their sleeping phase, both male and female msPs showed amplified acoustic startle responses versus non-selected Wistar counterparts. Furthermore, we observed that the level of startle responses was sex-dependent within the msP group, with female msPs displaying greater acoustic startle responses versus male msPs. Importantly, the enhanced anxiety-like behavior, lower average bout duration, and enhanced startle responses were not ameliorated by a single dose of mifepristone. Together, these findings suggest that the heightened magnitude of anxiety-related behaviors in msPs does not depend upon acute GR activation (see below for further discussion). Our findings provide a further step in understanding the role of the GR system in mediating anxiety-like states, particularly in models that display an innate sensitivity to negative affect.

A major finding of this study was that msPs display enhanced anxiety-like behaviors versus non-selected Wistar counterparts in an array of novel behavioral paradigms that are closely associated with stress disorders (e.g., PTSD). The latter finding is consistent with prior work from our laboratory demonstrating that msPs display enhanced anxiety-like behavior in the NIH and EPM [30] and marble-burying tasks [20]. Prior reports also have revealed that msPs display greater anxiety and depressive-like behaviors on numerous behavioral paradigms involving stress and anxiety. For example, msPs display more immobility on the forced swim test and more time on the corner zones of an open field arena, an indication that the magnitude of stress and anxiety is greater in msPs versus non-selected Wistars [12,13,14,30,31]. More recent studies in our laboratory have revealed that the high magnitude of stress and anxiety in msPs may relate to diminished HPA axis function, an effect that is unique to genetically-selected msPs [20]. The msP rats display impaired constraint of the HPA axis that normally curbs the stress response, and this effect is likely mediated in part by glucocorticoid signaling in the PVN [20]. Together these findings suggest that dysregulation of glucocorticoid signaling alters biological/brain systems in a manner that may contribute to the anxiety-like phenotype in msPs.

Since sleep disturbances and enhanced startle response are hallmark symptoms in the etiology of stress disorders, the present study examined for the first time whether genotypic differences may underlie changes during diurnal sleep maintenance and hyperarousal in msPs. These noninvasive and activity-based techniques correlate well with EEG-defined sleep studies [32], while startle responses can capture exaggerated hyperarousal similarly observed in human PTSD patients [33]. Importantly, we did not find genotype-dependent changes in sleep maintenance as defined by average bout duration, total sleep time, and number of sleep bouts. Notably, in the present study, the animals were not exposed to alcohol, which might be an important factor affecting the development of sleep disturbances that are often associated with AUD and anxiety disorders or PTSD symptoms. Indeed, msPs displayed enhanced acoustic startle responses as compared to their non-selected Wistar counterparts, suggesting that innate disrupted stress systems in msPs resulted in increased hyperarousal. Collectively, these data suggest that msPs are more vulnerable to stressful stimuli.

To explore the role of the glucocorticoid system in msPs, we employed acute systemic administration of mifepristone to reduce the levels of stress and anxiety across our battery of behavioral tests. We found that mifepristone administration produced no beneficial effects in anxiety-like behavior in the NIH paradigm. Also, mifepristone did not significantly restore sleep disturbances and hyperarousal to the level of healthy, non-selected Wistar controls. These findings are surprising since it has been well documented that a similar dose range of mifepristone reliably decreases alcohol-related behaviors in dependent Wistar rats (60 mg/kg) [21,34] and have anxiety alleviating behavioral effects in male rats (20 mg/kg, [35]; 120 mg/kg, [36]). There are likely a few possibilities to explain these findings. First, much work has revealed that prior exposure to physical (i.e., social defeat; [37]) or pharmacological (i.e., yohimbine; [25]) induction of stress is required for mifepristone to produce alleviating effects on anxiety in rats. This stress induction approach prior to mifepristone administration increases circulating stress hormone release that may increase the effectiveness of mifepristone binding activity to ameliorate anxiety. Second, since msPs contain a blunted stress response that is innate, an acute administration of mifepristone may not be sufficient to ameliorate the heightened levels of stress and anxiety. Indeed, repeated daily administration of mifepristone restores depressive-like behavior following chronic defeat stress in mice [37], while chronic mifepristone treatment also prevents escalation of alcohol self-administration over time in dependent rats [23]. In addition, acute versus chronic mifepristone treatment would impact non-genomic versus genomic GR signaling (respectively), likely representing differential mechanisms of action [38]. It should also be noted that mifepristone has demonstrated clinical efficacy in patients suffering from psychotic depression (reviewed in [39]). As such, to detect beneficial effects of mifepristone, future studies may involve chronic treatment regimens prior to behavioral assessments, as well as additional measures of negative affect. Lastly, prior work has revealed that msPs biochemically display impaired activation of the stress response, an effect largely mediated via glucocorticoid signaling in the CeA [20]. Specifically, phosphorylation of glucocorticoid receptor within the CeA is increased in male msPs versus non-selected Wistar rats and decreased in females [20]. The latter finding suggests that the regulation of GR signaling is compromised in the brains of msP rats, a neuroadaptation that may prevent the therapeutic effects of mifepristone administration in our experimental animal groups. Future studies will examine the mechanistic underpinnings of GR signaling on synaptic functions in the CeA.

While extensive work has focused on male msPs, there are only a few published reports studying the role of sex differences in modulating the anxiety-like predisposition in msPs [12,20,40]. Thus, the present study assessed the contribution of sex differences in promoting stress-related behaviors in a genotype-dependent manner. We found that males, regardless of genotype, generally display a suppression of food intake as compared to females during novelty stress. In addition, female msPs display a significant increase in startle response during the earliest and most intense audible trial as compared to their respective non-selected Wistar counterparts as well as to male msPs. Consistent with these findings, female msPs display higher amounts of alcohol consumption as compared to their respective Wistars counterparts as well as male msPs [12]. However, the latter report also revealed that both male and female msPs display similar levels of stress and anxiety in tasks involving forced swim and footshock procedures following chronic alcohol exposure [12]. This discrepancy between the latter report and our findings may be due to differences in the subjective effects of alcohol relative to alcohol-naïve states.

It is important to note that non-selectivity of mifepristone may exert several non-GR-associated actions. Mifepristone is known to be a competitive progesterone receptor antagonist, and progesterone may also serve to modulate anxiety-like behavior [41,42]. Thus, a possible explanation is that the effects of progesterone may contribute to the innate anxiety phenotype in msPs in a manner that prevents the therapeutic effects of mifepristone. Indeed, there is evidence suggesting that low levels of progesterone are correlated with greater anxiety-like behavior and corticosterone plasma levels [43]. Future work is needed to study the effects of mifepristone during specific phases of the estrous cycle, particularly in females who are more vulnerable to stress.

Overall, the present study revealed that both male and female msPs display elevated anxiety-like behavior versus their non-selected Wistar counterparts. In particular, female msPs display greater startle responses versus male msPs, suggesting that responses to stressful stimuli are sex-dependent. Our findings also indicate that mifepristone does not alleviate the innate anxiety-like profile of this genetically-selected rat model. One might expect that GR receptor antagonism would alleviate pre-existing anxiety-like traits in msPs. However, this drug may instead function to normalize the recovery after acute stress versus inherited stress-related traits. Furthermore, the purpose of this study was to determine the acute, non-genomic actions of mifepristone on anxiety-like behaviors since previous reports found that acute mifepristone reduced alcohol self-administration in dependent rats [21], suppressed yohimbine stress-induced reinstatement of alcohol-seeking [25], as well as acutely decreased negative affect produced by chronic stress in mice with high-trait anxiety [27]. Thus, future studies are required to examine the delayed genomic effects following chronic mifepristone administration. Future work is also needed to fully understand the stress systems in relation to stress hormone fluctuations as well as changes in circadian rhythms, in addition to potential biomarkers that interact with glucocorticoid-GR systems for optimal administration of mifepristone.

## 4. Materials and Methods

### 4.1. Animals

A total of N = 143 rats were used in this study. Adult male (*n* = 40, ~450 g) and female (*n* = 34, ~250 g) msP rats were bred at The Scripps Research Institute (La Jolla, CA, USA) from a colony obtained from the University of Camerino (Camerino, Italy). For genotypic comparisons, we used adult male (*n* = 35, ~450 g) and female (*n* = 34, ~250 g) Wistar rats (Envigo, Indianapolis, IN, USA) from which the msP line was generated. Rats were housed on a 12 h reverse light/dark cycle (lights off at 8:00 a.m.), with food and water available *ad libitum*. The rats were pair-housed, separated by a perforated clear plexiglass divider to habituate them to the behavioral test conditions while also reducing isolation stress [44]. The experimental groups consisted of rats that were randomly selected, and simple randomization for treatment groups condition occurred prior to the start of the experiments via a number labeling system for each rat. Then, the rats were arbitrarily assigned to different treatment groups regardless of body weight. We conducted all procedures in accordance with the National Institutes of Health Guide for the Care and Use of Laboratory Animals and with The Scripps Research Institute Institutional Animal Care and Use Committee policies.

### 4.2. Drug Preparation and Treatment

Rats were injected intraperitoneally (i.p.) with a single dose (60 mg/kg; 1 mL/kg) of mifepristone (Cayman Chemical, Ann Arbor, MI, USA) or vehicle (propylene glycol) 90 min prior to each behavioral test. At the used dose and injection volume, mifepristone is characterized by poor solubility in aqueous vehicles. To improve the solubility, 100% propylene glycol [45] was slowly added to the compound while mixing with a magnetic stir bar. The 60 mg/kg dose and 90 min pretreatment were chosen based on previously published reports demonstrating that the acute systemic administration of this dose of mifepristone reduces alcohol reinforcement [21], and doses ranging between 20 mg/kg and 120 mg/kg have an anxiolytic effect in male rats [35,36].

### 4.3. Novelty-Induced Hypophagia (NIH)

Exposure to novel environments elicits a stressful reaction in rodents that can interfere with normal behavior, including food consumption [46,47]. Here rats were monitored for anxiety-like behavior using hypophagia procedures as previously published [48]. The animals received home cage exposure to a novel palatable food (50% sucrose, chocolate-flavored pellets, 45 mg, 5TUL, Test Diets, St. Louis, MO, USA) 24 h before being tested. The rats were acclimated to their housing room during their dark phase (red lights). Following exposure to the novel palatable food, rats were monitored by the experimenter to confirm that each rat tasted the novel food. The following day, the rats were treated with mifepristone (60 mg/kg, 1 mL/kg, i.p.) or vehicle and were left undisturbed for 90 min prior to evaluation under novel testing conditions that are perceived to be stressful (i.e., white lights on, unfamiliar double-size cage, white noise). We measured the latency for the rats to consume the chocolate pellets and the total intake in an unfamiliar environment over a 10 min trial.

### 4.4. Comprehensive Lab Animal Monitoring System (CLAMS)

Since stress and anxiety are often accompanied by sleep disturbances [49], we assessed sleep patterns in a comprehensive lab animal monitoring system (CLAMS, Columbus Instruments, Columbus, OH, USA). Such noninvasive, activity-based measurements correlate well with EEG-defined sleep [32,44]. We used OXYmax–CLAMS units to interpret each rat’s sleep parameters from photocell-defined motor activity across the first 11 h of their 12 h light phase. Rats were placed in the single units (32 × 20 × 19 cm) 12 h prior to testing to allow them to acclimatize. Each CLAMS chamber was equipped with a water sipper and tray that provided *ad libitum* access to food. Twenty-four photobeams were used to detect horizontal or vertical movement and were located 2.5 cm apart, at 9 cm and 14 cm above the floor. CLAMS sleep detection works in time periods called “epochs”. Sleeping epochs were defined as no more than one photocell interruption during a 60 sec epoch, and sleep bouts were defined as successive strings of such epochs. We extracted and analyzed the average duration of the sleeping bouts (min), the number of sleeping bouts and the total sleeping time (min) throughout the inactive phase.

### 4.5. Acoustic Startle

Exaggerated acoustic startle responses are present in patients with PTSD and indicate hyperarousal [44]. Startle reflexes were measured in four identical startle response systems (SR-LAB, San Diego Instruments, San Diego, CA, USA) consisting of a nonrestrictive Plexiglas cylinder (13 cm inner diameter, 25 cm length for males; 9 cm inner diameter, 20 cm length for females) mounted on a Plexiglas platform and placed in a dark, ventilated, sound-attenuated chamber. The movements were detected and measured by a piezoelectric element mounted under each cylinder. A dynamic calibration system was used to ensure comparable startle magnitudes across the four devices. Throughout the session, the startle system delivered a constant background white noise of 68 dB. Startle stimuli were presented through a high-frequency speaker located above the startle chambers and lasted for 30 msec. Startle magnitudes were sampled each millisecond during a period of 100 msec, beginning at the onset of the startle stimulus. Startle response was defined as the peak response during this 100 msec period. During a 30 min session, 75 trials were presented in a pseudorandom order. The SR-LAB startle response system measured startle responses to acoustic stimuli (80–120 dB) and no-stimulus control trials. The test session began with a 5 min acclimation period followed by four consecutive blocks of test trials. Block 1 and 4 consisted of six startle 120 dB stimulus-alone trials. Prepulse inhibition was tested in block 2 by interspersing non-prepulsed 120 dB trials with six 120 dB trials that were prepulsed with an 80 dB tone by 70 msec. Block 3 consisted of trials of varying intensity (80, 85, 90, 95, 100, 105 dB), each one presented six times in a randomized order. Between each block, three no-stimulus trials were included, during which only the background noise was presented.

### 4.6. Statistical Analyses

NIH, CLAMS, and acoustic startle were analyzed using separate two-way analyses of variance (ANOVA) with treatment (vehicle versus mifepristone) and genotype (Wistar versus msP) as between-subject factors. Significant interaction effects were followed by Fisher’s LSD-protected post hoc tests. For acoustic startle data containing repeated stimulus intensities, a mixed model three-way ANOVA was used with genotype and drug treatment as between subjects’ factors and levels of intensity as within-subject factor. To investigate the role of sex differences, a separate level of analysis using similar two-way ANOVAs was included with sex (male versus female) and genotype (Wistar versus msP) as between-subject factors. The rationale for this analytical approach is based on our hypothesis regarding drug treatment effects separately within each sex and based on prior work [20]. Each sex was tested on separate days with experimenters blind to the subjects’ treatment condition. All data are presented as mean ± standard error of the mean (SEM). The significance level was determined at *p* < 0.05. All statistical analyses were performed on SPSS V26 (IBM Corporation, Armonk, NY, USA), and all graphs were generated using Prism V8 (GraphPad, San Diego, CA, USA). For a complete description of the individual experiments’ statistical analysis, please see Supplementary Tables S1–S4.

## Data Availability

The data presented in this study are available upon request from the corresponding author.

## Supplementary Materials

The following are available online at https://www.mdpi.com/1422-0067/22/6/3095/s1.
