# Supplementary material for: Glucocorticoid Receptor Antagonist Mifepristone Does Not Alter Innate Anxiety-Like Behavior in Genetically-Selected Marchigian Sardinian (msP) Rats
Source: Int J Mol Sci. 2021 Mar 18;22(6):3095. doi: 10.3390/ijms22063095 (PMC8003048; doi:10.3390/ijms22063095)
Supplement: Supplementary file 1 [file ijms-22-03095-s001.pdf]

Table S1. Summary of all statistical analysis for the NIH test.

Figure	Analysis	Sex	Dependent variable	Factors	Statistical value
1A	2-way ANOVA	Male	Latency to eat	Drug (vehicle vs mifepristone) × Genotype (Wistar vs msP)	Drug: $F_{(1,40)} = 0.07$, $p = 0.780$<br>***Genotype: $F_{(1,40)} = 42.52$, $p = 0.001$<br>Drug × Genotype: $F_{(1,40)} = 1.69$, $p = 0.201$
1B	2-way ANOVA	Male	Pellet intake	Drug (vehicle vs mifepristone) × Genotype (Wistar vs msP)	Drug: $F_{(1,40)} = 0.48$, $p = 0.490$<br>***Genotype: $F_{(1,40)} = 49.48$, $p = 0.0001$<br>Drug × Genotype: $F_{(1,40)} = 0.31$, $p = 0.576$
1C	2-way ANOVA	Female	Latency to eat	Drug (vehicle vs mifepristone) × Genotype (Wistar vs msP)	Drug: $F_{(1,32)} = 0.195$, $p = 0.662$<br>***Genotype: $F_{(1,32)} = 12.10$, $p = 0.001$<br>Drug × Genotype: $F_{(1,32)} = 0.28$, $p = 0.600$
1D	2-way ANOVA	Female	Pellet intake	Drug (vehicle vs mifepristone) × Genotype (Wistar vs msP)	Drug: $F_{(1,32)} = 0.07$, $p = 0.783$<br>***Genotype: $F_{(1,32)} = 16.69$, $p = 0.0001$<br>Drug × Genotype: $F_{(1,32)} = 1.41$, $p = 0.244$
1E	2-way ANOVA	Male x Female	Latency to eat	Sex (male vs female) × Genotype (Wistar vs msP)	Sex: $F_{(1,35)} = 0.27$, $p = 0.601$<br>Genotype: $F_{(1,35)} = 34.65$, $p = 0.0001$<br>Sex × Genotype: $F_{(1,35)} = 0.96$, $p = 0.332$
1F	2-way ANOVA	Male x Female	Pellet intake	Sex (male vs female) × Genotype (Wistar vs msP)	@Sex: $F_{(1,35)} = 4.91$, $p = 0.048$<br>Genotype: $F_{(1,35)} = 10.91$, $p = 0.002$<br>Sex × Genotype: $F_{(1,35)} = 0.01$, $p = 0.90$

Note: Significant changes are reflected as *$p \leq 0.05$, **$p \leq 0.01$, ***$p \leq 0.001$ or sex effects as @$p \leq 0.05$.

Table S2. Summary of all statistical analysis for the CLAMS test

Figure	Analysis	Sex	Dependent variable	Factors	Statistical value
2A	2-way ANOVA	Male	Average bout duration	Drug (vehicle vs mifepristone) × Genotype (Wistar vs msP)	Drug: $F_{(1,27)} = 3.06$, $p = 0.092$<br>*Genotype: $F_{(1,27)} = 4.92$, $p = 0.035$<br>Drug × Genotype: $F_{(1,27)} = 1.10$, $p = 0.303$
2B	2-way ANOVA	Male	Total sleep time	Drug (vehicle vs mifepristone) × Genotype (Wistar vs msP)	Drug: $F_{(1,27)} = 2.42$, $p = 0.131$<br>Genotype: $F_{(1,27)} = 0.51$, $p = 0.479$<br>Drug × Genotype: $F_{(1,27)} = 3.11$, $p = 0.089$
2C	2-way ANOVA	Male	Number of sleep bouts	Drug (vehicle vs mifepristone) × Genotype (Wistar vs msP)	Drug: $F_{(1,27)} = 4.08$, $p = 0.053$<br>Genotype: $F_{(1,27)} = 4.13$, $p = 0.052$<br>Drug × Genotype: $F_{(1,27)} = 1.10$, $p = 0.210$
2D	2-way ANOVA	Female	Average bout duration	Drug (vehicle vs mifepristone) × Genotype (Wistar vs msP)	Drug: $F_{(1,28)} = 1.21$, $p = 0.280$<br>Genotype: $F_{(1,28)} = 0.07$, $p = 0.789$<br>Drug × Genotype: $F_{(1,28)} = 2.49$, $p = 0.126$
2E	2-way ANOVA	Female	Total sleep time	Drug (vehicle vs mifepristone) × Genotype (Wistar vs msP)	Drug: $F_{(1,28)} = 0.38$, $p = 0.539$<br>Genotype: $F_{(1,28)} = 2.23$, $p = 0.146$<br>Drug × Genotype: $F_{(1,28)} = 9.36$, $p = 0.005$
2F	2-way ANOVA	Female	Number of sleep bouts	Drug (vehicle vs mifepristone) × Genotype (Wistar vs msP)	Drug: $F_{(1,28)} = 0.76$, $p = 0.388$<br>Genotype: $F_{(1,28)} = 0.02$, $p = 0.872$<br>Drug × Genotype: $F_{(1,28)} = 1.58$, $p = 0.219$
2G	2-way ANOVA	Male x Female	Average bout duration	Sex (male vs female) × Genotype (Wistar vs msP)	Sex: $F_{(1,27)} = 1.23$, $p = 0.276$<br>Genotype: $F_{(1,27)} = 5.70$, $p = 0.024$<br>Sex × Genotype: $F_{(1,27)} = 2.61$, $p = 0.117$
2H	2-way ANOVA	Male x Female	Total sleep time	Sex (male vs female) × Genotype (Wistar vs msP)	Sex: $F_{(1,27)} = 0.89$, $p = 0.354$<br>Genotype: $F_{(1,27)} = 4.24$, $p = 0.049$<br>Sex × Genotype: $F_{(1,27)} = 0.46$, $p = 0.502$
2I	2-way ANOVA	Male x Female	Number of sleep bouts	Sex (male vs female) × Genotype (Wistar vs msP)	Sex: $F_{(1,27)} = 0.003$, $p = 0.954$<br>Genotype: $F_{(1,27)} = 4.72$, $p = 0.039$<br>Sex × Genotype: $F_{(1,27)} = 1.11$, $p = 0.300$

Note: Significant changes are reflected as *$p \leq 0.05$.

Table S3. Summary of statistical analysis for the acoustic startle test

Figure	Analysis	Sex	Dependent variable	Factors	Statistical value
3A	2-way ANOVA	Male	120 dB T1	Drug (vehicle vs mifepristone) × Genotype (Wistar vs msP)	Drug: $F_{(1,26)} = 1.60$, $p = 0.217$<br>Genotype: $F_{(1,26)} = 0.84$, $p = 0.366$<br>Drug × Genotype: $F_{(1,26)} = 0.68$, $p = 0.414$
3B	2-way ANOVA	Male	120 dB T2-6	Drug (vehicle vs mifepristone) × Genotype (Wistar vs msP)	Drug: $F_{(1,26)} = 0.55$, $p = 0.463$<br>**Genotype: $F_{(1,26)} = 10.56$, $p = 0.003$<br>Drug × Genotype: $F_{(1,26)} = 0.98$, $p = 0.330$
3C	2-way ANOVA	Male	120 dB final block	Drug (vehicle vs mifepristone) × Genotype (Wistar vs msP)	Drug: $F_{(1,26)} = 0.10$, $p = 0.751$<br>***Genotype: $F_{(1,26)} = 14.51$, $p = 0.001$<br>Drug × Genotype: $F_{(1,26)} = 0.66$, $p = 0.424$
3D	2-way ANOVA	Male	Prepulse inhibition	Drug (vehicle vs mifepristone) × Genotype (Wistar vs msP)	Drug: $F_{(1,26)} = 1.19$, $p = 0.284$<br>**Genotype: $F_{(1,26)} = 9.32$, $p = 0.005$<br>Drug × Genotype: $F_{(1,26)} = 1.24$, $p = 0.274$
3E	3-way ANOVA	Male	80-105 dB	Drug (vehicle vs mifepristone) × Genotype (Wistar vs msP) x Intensity (80-105db)	Intensity: $F_{(5,130)} = 70.82$, $p = 0.0001$<br>Drug × Intensity: $F_{(5,130)} = 1.08$, $p = 0.373$<br>Genotype × Intensity: $F_{(5,130)} = 9.25$, $p = 0.001$<br>Drug × Genotype × Intensity: $F_{(5,130)} = 0.57$, $p = 0.720$
3F	2-way ANOVA	Female	120 dB T1	Drug (vehicle vs mifepristone) × Genotype (Wistar vs msP)	Drug: $F_{(1,28)} = 2.15$, $p = 0.153$<br>**Genotype: $F_{(1,28)} = 8.89$, $p = 0.006$<br>Drug × Genotype: $F_{(1,28)} = 0.23$, $p = 0.630$
3G	2-way ANOVA	Female	120 dB T2-6	Drug (vehicle vs mifepristone) × Genotype (Wistar vs msP)	Drug: $F_{(1,28)} = 1.43$, $p = 0.241$<br>Genotype: $F_{(1,28)} = 4.13$, $p = 0.052$<br>Drug × Genotype: $F_{(1,28)} = 2.26$, $p = 0.143$
3H	2-way ANOVA	Female	120 dB final block	Drug (vehicle vs mifepristone) × Genotype (Wistar vs msP)	Drug: $F_{(1,28)} = 0.54$, $p = 0.466$<br>*Genotype: $F_{(1,28)} = 5.37$, $p = 0.028$<br>Drug × Genotype: $F_{(1,28)} = 1.20$, $p = 0.282$
3I	2-way ANOVA	Female	Prepulse inhibition	Drug (vehicle vs mifepristone) × Genotype (Wistar vs msP)	Drug: $F_{(1,28)} = 0.75$, $p = 0.392$<br>*Genotype: $F_{(1,28)} = 7.14$, $p = 0.012$<br>Drug × Genotype: $F_{(1,28)} = 0.64$, $p = 0.428$
3J	3-way ANOVA	Female	80-105 dB	Drug (vehicle vs mifepristone) × Genotype (Wistar vs msP) x Intensity (80-105db)	Intensity: $F_{(5,140)} = 34.60$, $p = 0.0001$<br>Drug × Intensity: $F_{(5,140)} = 0.58$, $p = 0.711$<br>Genotype × Intensity: $F_{(5,140)} = 4.88$, $p = 0.026$<br>Drug × Genotype × Intensity: $F_{(5,140)} = 1.72$, $p = 0.198$

Note: Significant changes are reflected as *$p \leq 0.05$, **$p \leq 0.01$, ***$p \leq 0.001$.

Table S4. Summary of statistical analysis for sex differences for the acoustic startle test

Figure	Analysis	Sex	Dependent variable	Factors	Statistical value
3K	2-way ANOVA	Male x Female	120 dB T1	Sex (male vs female) × Genotype (Wistar vs msP)	Sex: $F_{(1,26)} = 12.08$, $p = 0.002$<br>Genotype: $F_{(1,26)} = 6.09$, $p = 0.020$<br>Sex × Genotype: $F_{(1,26)} = 4.81$, $p = 0.037$
3L	2-way ANOVA	Male x Female	120 dB T2-6	Sex (male vs female) × Genotype (Wistar vs msP)	@@@Sex: $F_{(1,26)} = 18.14$, $p = 0.0001$<br>Genotype: $F_{(1,26)} = 2.15$, $p = 0.154$<br>Sex × Genotype: $F_{(1,26)} = 0.09$, $p = 0.761$
3M	2-way ANOVA	Male x Female	120 dB final block	Sex (male vs female) × Genotype (Wistar vs msP)	@@@Sex: $F_{(1,26)} = 18.20$, $p = 0.0001$<br>Genotype: $F_{(1,26)} = 11.15$, $p = 0.003$<br>Sex × Genotype: $F_{(1,26)} = 1.30$, $p = 0.264$
3N	2-way ANOVA	Male x Female	Prepulse inhibition	Sex (male vs female) × Genotype (Wistar vs msP)	@Sex: $F_{(1,26)} = 5.61$, $p = 0.025$<br>Genotype: $F_{(1,26)} = 8.79$, $p = 0.006$<br>Sex × Genotype: $F_{(1,26)} = 0.02$, $p = 0.889$
3O	3-way ANOVA	Male x Female	80-105 dB	Sex (male vs female) × Genotype (Wistar vs msP)	@@@Sex: $F_{(1,26)} = 20.02$, $p = 0.0001$<br>Intensity: $F_{(5,130)} = 53.99$, $p = 0.001$<br>Sex × Intensity: $F_{(5,130)} = 12.66$, $p = 0.0001$<br>Genotype × Intensity: $F_{(5,130)} = 3.36$, $p = 0.007$<br>Sex × Genotype × Intensity: $F_{(5,130)} = 0.51$, $p = 0.767$

Note: Significant changes are reflected as @$p \leq 0.05$, @@$p \leq 0.01$, @@@$p \leq 0.001$.
